# Comparative neuroprotective effects of antidiabetic medications on Parkinson's disease risk: a Bayesian network meta-analysis

**DOI:** 10.7189/jogh.16.04187

**Published:** 2026-08-21

**Authors:** Soo Hyeon Lee, Yujin Rho, Sangyoon Lee, Sooyoung Shin, Yeo Jin Choi

**Affiliations:** 1 Department of Regulatory Science, Graduate School, Kyung Hee University, Seoul, Republic of Korea; 2 Institute of Regulatory Innovation through Science, Kyung Hee University, Seoul, Republic of Korea; 3 College of Pharmacy and Institute of Integrated Pharmaceutical Sciences, Kyung Hee University, Seoul, Republic of Korea; 4 Department of Pharmacy, Union Hospital, Terre Haute, Indiana, USA; 5 Department of Pharmacy, College of Pharmacy, Ajou University, Suwon, Republic of Korea; 6 Institute of Pharmaceutical Science and Technology, Ajou University, Suwon, Republic of Korea

**Keywords:** Parkinson’s disease, diabetes mellitus, sodium-glucose co-transporter-2 inhibitor, metformin, cardiovascular disease, GLP-1 receptor agonist

## Abstract

**Background:**

Diabetes mellitus is recognised as a risk factor for Parkinson’s disease (PD); however, comparative evidence regarding PD risk across antidiabetic drug classes remains limited and inconsistent. We aimed to compare the risk of PD across different antidiabetic classes, with stratified analyses by age, sex, and cardiovascular disease (CVD) status.

**Methods:**

We searched the Cochrane Library, Embase, and PubMed until August 2025 for studies assessing the association between antidiabetic drugs and PD incidence. We performed a Bayesian network meta-analysis, estimating effect sizes as risk ratios with 95% credible intervals. We performed prespecified subgroup analyses according to age, sex, and CVD status.

**Results:**

We included nine observational cohort studies, totalling 712 287 patients. No antidiabetic class demonstrated a statistically significant difference in PD risk. Rank probability analyses suggested that sodium-glucose co-transporter-2 inhibitors (SGLT2i) tended to rank lowest in PD risk among older adults (≥75 years), while glucagon-like peptide-1 receptor agonist (GLP-1RA) ranked lowest in patients aged <75 years. In patients with CVD, metformin showed relatively higher-ranking probabilities for PD risk compared with SGLT2i. In contrast, metformin, sulfonylureas, and α-glucosidase inhibitors were consistently associated with higher ranking probabilities for PD risk compared to newer agents.

**Conclusions:**

Our analysis suggests that antidiabetic drugs may exert effects beyond glycaemic control and could influence neurodegenerative risk. Furthermore, SGLT2i and GLP-1RA were consistently associated with lower PD risk, suggesting potential neuroprotective effects. While our results indicate the potential importance of antidiabetic drug selection in patients at risk for neurodegenerative diseases, further large-scale, controlled studies should validate these associations and clarify potential mechanisms.

**Registration:**

PROSPERO: CRD420251130232

Parkinson disease (PD) is a common neurodegenerative disease that affects 2–3% of the global older population [1,2]. Estimates suggest that the number of affected individuals will exceed 25 million by 2050, raising concerns that PD mortality may surpass that of cancer [3]. The condition is characterised by motor dysfunction, cognitive decline, sleep disturbance, and psychiatric disturbances, resulting in substantial functional impairment and negatively impacting patients’ quality of life [1,4].

As current treatments primarily provide symptomatic relief without altering the underlying disease course, identifying modifiable risk factors for PD and developing effective preventive strategies remain crucial [5].

Diabetes is increasingly recognised as a metabolic risk factor for neurodegeneration, with accumulating evidence indicating it is associated with an elevated risk of PD [6]. Several biological mechanisms have been implicated in the increased risk of PD among individuals with diabetes. For example, chronic hyperglycaemia and insulin resistance promote oxidative stress, mitochondrial dysfunction, and neuroinflammation, thereby increasing vulnerability to dopaminergic neuronal loss [7,8]. Neuroinflammation represents a shared pathogenic pathway, whereby α-synuclein-mediated microglial activation induces neuronal death through inflammatory cytokines such as interleukin-2 and tumour necrosis factor-α [9,10]. These mechanisms provide a biological explanation for the association between diabetes and PD and suggest that improving glycaemic control and reducing insulin resistance may mitigate the risk of developing PD [11].

Relatedly, prior studies have suggested that antidiabetic agents may influence the risk of individuals developing Alzheimer's disease [12]. Glucagon-like peptide-1 receptor agonists (GLP-1RAs) have demonstrated neuroprotective effects through modulation of neuroinflammation, improved mitochondrial function, and enhanced neuronal survival [13], with recent evidence suggesting symptomatic benefits in PD [14]. Similarly, thiazolidinediones (TZD) may reduce neuroinflammation *via* peroxisome proliferator-activated receptor-γ activation, metformin may enhance AMP-activated protein kinase-mediated insulin signalling and mitochondrial function, and sodium-glucose co-transporter-2 inhibitor (SGLT2i) may attenuate oxidative stress and neurovascular dysfunction, all of which are implicated in neurodegeneration [13]. However, comparative evidence across antidiabetic drug classes remains limited and inconsistent. Therefore, we aimed to compare the risk of incident PD across antidiabetic drug classes in patients with type 2 diabetes mellitus using a Bayesian network meta-analysis and to identify high-risk subgroups based on age, sex, and comorbidities.

## METHODS

### Database search and study selection

We registered our protocol in PROSPERO (CRD420251130232) and followed the PRISMA extension for network meta-analyses in reporting our findings [15]. We searched Cochrane Library, Embase, and PubMed from inception to August 2025 using prespecified MeSH and keywords, such as diabetes mellitus, PD, Parkinson, hypoglycaemic agents, antidiabetics, insulin, dipeptidyl-peptidase-4 inhibitor (DPP4i), SGLT2i, sulfonylurea, metformin, TZD, GLP-1RA, and meglitinide (Table S1 in the **Online Supplementary Document**). We included both generic and brand names of antidiabetic agents. We limited the searches to human populations and studies published in English.

We selected studies based on prespecified patient, intervention, comparator, outcomes, and study design criteria. Specifically, we included research on patients with diabetes receiving antidiabetic treatment, where either the intervention and comparator involved antidiabetic medication classes, and where the outcome was incident PD, diagnosed using ICD codes. Eligible study designs included randomised controlled trials (RCTs) and observational cohort studies. We only considered studies reported in English whose full text we could access.

We excluded duplicated publications, reviews, case reports, case-control studies (because of inherent bias and temporal ambiguity), study protocols, conference abstracts, unpublished manuscripts, comparisons between diabetes *vs.* non-diabetes populations, comparisons of users *vs.* non-users within the same drug class (due to limited relevance for between-class comparisons and potential confounding by indication), studies with inadequate adjustment for baseline characteristics (*e.g.* absence of propensity score methods or multivariable adjustment for key confounders, such as age and sex), and studies evaluating combination antidiabetic regimens.

Two reviewers (SHL and YR) independently extracted study characteristics (author, year of publication, country, study design), study population, study intervention and comparators, study outcomes, and follow-up duration. We manually screened reference lists of eligible articles and relevant reviews to identify additional studies.

### Risk of bias

As we only identified observational cohort studies that met our inclusion criteria, two reviewers (SHL and YR) independently assessed risk of bias using the ROBINS-I tool, which comprises the following domains: confounding, participant selection, intervention classification, deviations from intended interventions, missing data, outcome measurement, and selective reporting [16].

### Statistical analysis

We performed a Bayesian network meta-analysis using Markov Chain Monte Carlo simulation with ‘gemtc’ and ‘rjags’ packages in *R*. We specified non-informative prior distributions according to the default ‘gemtc’ package settings. We assigned vague normal priors (mean = 0; variance = 10,000) to treatment effects, described using log risk ratios (RRs), and modelled between-study heterogeneity using a uniform prior standard deviation (0–5). Direct and indirect comparisons across antidiabetic classes were synthesised to estimate RRs with 95% credible intervals (CrIs). Then, we examined network geometry using network plots to visualise evidence distribution and network connectivity [17]. Here, local consistency was evaluated using node-splitting methods by comparing direct and indirect estimates. Inconsistency was considered present when the 95% CrI for the differences between direct and indirect estimates included the null value [17]. We evaluated the transitivity assumption by examining the comparability of potential effect modifiers across treatment comparisons, including age, sex, cardiovascular disease (CVD) status, renal disease, follow-up duration, and study design (Table S2 in the **Online Supplementary Document**). We did not identify a clinically meaningful imbalance, supporting the validity of indirect comparisons.

We assessed the between-study heterogeneity using the between-study standard deviation derived from the Bayesian random-effects model, reporting *I*^2^ as a supplementary measure. Both fixed-effect and random-effects Bayesian models were implemented, with the random-effects model selected based on the deviance information criterion due to observed between-study heterogeneity [17]. We assessed model convergence through visual inspection of trace plots and the Gelman–Rubin diagnostic, with all monitored parameters demonstrating satisfactory convergence (R̂ < 1.01). We used SGLT2i as the reference group due to its established cardiovascular and renal benefits and emerging evidence of reduced neurodegenerative risk, including PD and Alzheimer disease [13,18–20]. Recent guidelines recognise SGLT2 inhibitors as first-line options [21]. The choice of reference treatment does not affect relative effect estimates or treatment rankings in network meta-analysis.

We evaluated publication bias with Egger’s test and Begg’s funnel plot, with *P* > 0.05 and a symmetric funnel plot indicating low risk of publication bias [22]. Treatment ranking probabilities were summarised using the surface under the cumulative ranking curve (SUCRA), with higher SUCRA values indicating a lower PD risk. We conducted subgroup analyses to investigate the clinical impact of patient-specific factors, including age (<75 and ≥75 years), sex, and CVD history using study-level stratified data reported in the original studies within the Bayesian network meta-analysis. We performed sensitivity analyses by restricting the analysis to studies with a follow-up duration of ≥2 years to assess the robustness of the findings.

We used *R*, version 4.1.0 (R Core Team, Vienna, Austria) for all analyses. We assessed statistical significance using 95% CrIs, considering estimates statistically meaningful when the CrI did not include the null value.

## RESULTS

### Characteristics of included studies

**Figure 1:**
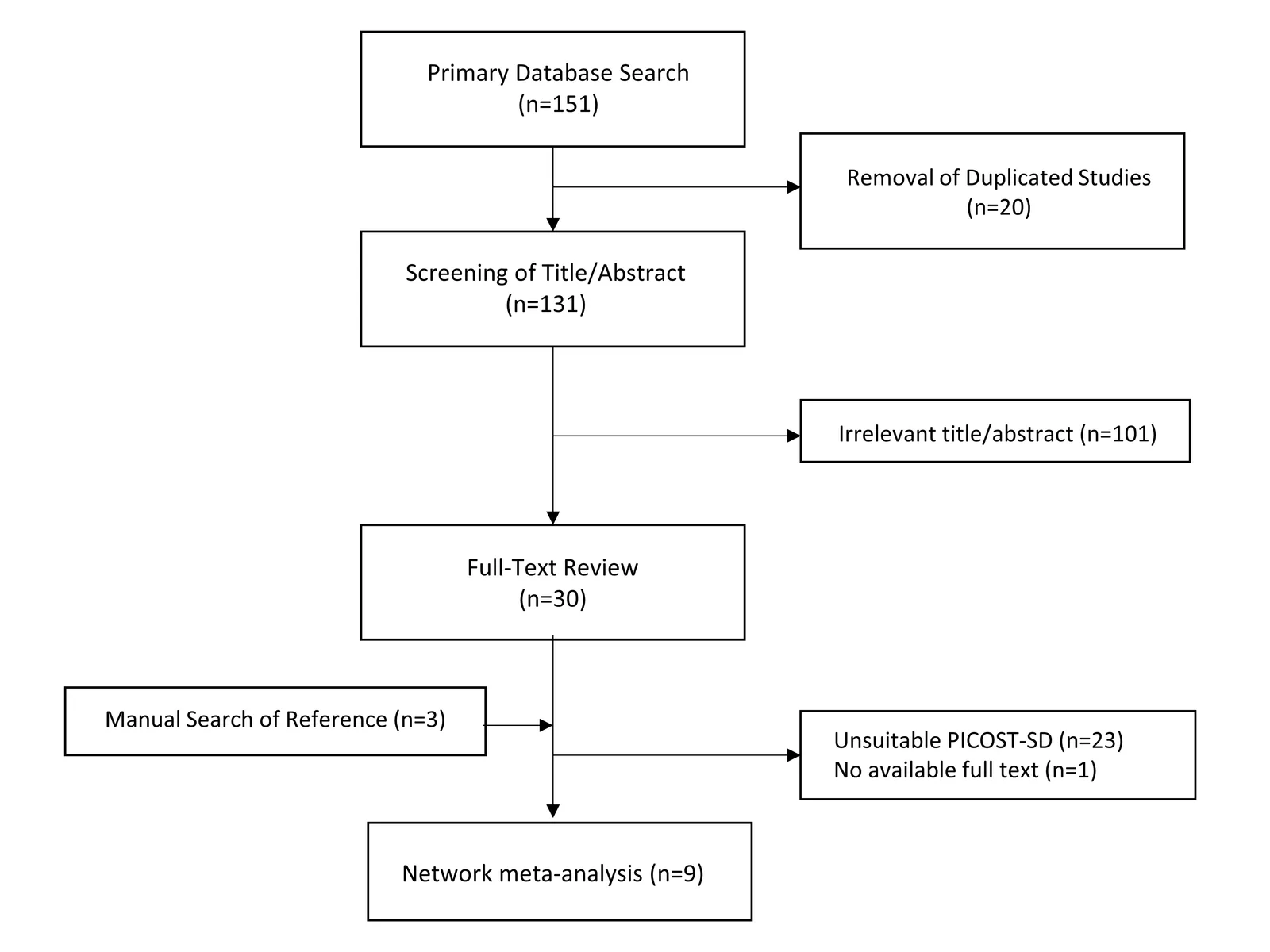
Database search and study selection process.

The initial database search and study selection process yielded 151 studies, of which 30 were eligible for full-text review after title/abstract screening (**Figure 1**). Of these 30 studies, 6 were eligible for inclusion, while 3 more studies were identified from a manual search of their references, leaving 9 studies comprising 712 287 participants for analysis [23–31]. All of the included studies had a retrospective cohort design, as we did not identify RCTs directly comparing the risk of developing PD across antidiabetic drug classes (**Table 1**). Geographically, one was multinational [25], three were conducted in the USA [26–28], two in China [24,30], and one in Norway [23], Taiwan [29], and the UK [31], respectively. Seven antidiabetic regimens were included: α-glucosidase inhibitor (AGI) [30], DPP4i [24,26,27,29,31], GLP-1RA [27,31], metformin [23,25], SGLT2i [24–26], sulfonylurea [28,29], and TZD [23,28,30,31]. Four studies included diabetic patients aged ≥40 years [26-29], three of which specifically focused on older patients aged ≥65 years [26–28].

**Table 1:** Study characteristics

Study, year, reference	Study design (country)	Patient population	Exclusion criteria	Confounding adjustment methods	Index date	Exposure definition	Intervention and comparators	Outcomes and cumulative exposure	Follow-up period in years
Brauer *et al.* (2020) [31]	Longitudinal population-based cohort (UK)	Patients diagnosed with T2DM (n = 68 756)	Diagnosis of PD before the first prescription of antidiabetics or a record of secondary Parkinsonism	PS matching on comorbidities, BMI, drinking status, and time between first DM diagnosis	First prescription with dispensed medications (new user)	≥2 consecutive prescriptions for study medications	TZD (n = 21 175); DPP4i (n = 36 897), GLP-1RA (n = 10 684)	PD diagnosis; Cox regression with IPTW	3.6 (1.7–6.4)*
Brakedal *et al.* (2017) [23]	Retrospective cohort using the Norwegian Prescription Database (Norway)	Patients diagnosed with DM who used TZD or metformin from January 2005 to December 2014 (n = 102 745)	Individuals who received PD medications in the first 12 months after baseline	Time-dependent Cox regression analysis adjusted for sex and age	Date of first drug dispensing	≥2 consecutive prescriptions for study medications	TZD (n = 8396); Metformin (n = 94 349)	PD diagnosis; Time-dependent Cox regression model	6.94†
Tang *et al.* (2024) [27]	Population-based cohort using the USA Medicare administrative data (USA)	Older patients (≥66 years) with T2DM (n = 89 074)	Any form of Parkinsonism, Lewy body dementia, end-stage renal disease, and prior exposure to anti-PD medications	Stabilised IPTW on age, sex, race, income, comorbidity, and comedication	First prescription for GLP-1RA or DPP4i defined as without a previous prescription for either drug within the preceding year	Initiation of GLP-1RA or DPP4i	GLP-1RA (n = 30 091); DPP4i (n = 58 983)	PD diagnosis; Cox proportional hazards model, additional as-treated model	GLP-1RA: 1.54 (0.75–2.53); DPP4i: 1.74 (0.83–2.77)*
Guo *et al.* (2024) [26]	Retrospective cohort using a random sample of Medicare Part D beneficiaries from the Centres for Medicare and Medicaid Services from 2016–2020 (USA)	Older patients (≥65 years) with T2DM (n = 53 238)	Diagnosis of PD, parkinsonism conditions, or use of anti-PD medications at baseline	PS matching on age, number of antidiabetic medications at baseline, comorbidities, and year of enrollment	Date of the first prescription fill of an SGLT2i or a DPP4i, identified by not using either of these two drugs in the prior year	Initiation of SGLT2i or DPP4i	SGLT2i (n = 26 619); DPP4i (n = 26 619)	PD diagnosis; Cox proportional hazards model, Fine and Gray methods, Temporal analysis excluding those having a PD diagnosis within 1 year after the index date	NA
Connolly *et al.* (2015) [28]	Retrospective cohort from Medicare beneficiaries from January 1997 to December 2005 (USA)	Older patients (≥65 years) with T2DM (n = 10 450)	Patients who had a diagnosis of PD, extrapyramidal symptoms, or received an antiparkinsonian medication that was recorded in the data at any time before the index date	PS matching on age, sex, race, co-medications, number of hospitalisations, and comorbidities	First pharmacy dispensation of either a TZD or a sulfonylurea	New users; additional analysis on patients with ≥3 months of continuous use	Sulfonylurea (n = 5225); TZD (n = 5225)	PD diagnosis; ITT analysis and as-treated analysis	2.97†
Huang *et al.* (2024) [29]	Retrospective cohort using Taiwan’s HWDC from 2008–2016 (Taiwan)	Older patients (≥50 years) with T2DM diagnosis in 2009–2013 (n = 103 636)	Patients who had a PD diagnosis before DM, within one year of DM diagnosis	PS matching on gender, age, income level, and DM complications severity index	New DPP4i or sulfonylurea users after DM diagnosis	Initiation of DPP4i or sulfonylurea	DPP4i (n = 25 909); Sulfonylurea (n = 77 727)	PD diagnosis; Cox hazards model, HR adjusted through the Bonferroni adjustment test, additional analysis based on the defined daily dose	3†
Sun *et al.* (2025) [25]	TriNet X platform (142 healthcare organisations worldwide)	Adults (≥18 years) diagnosed with DM between 20 January 2005 and 20 January 2025, and initiated on treatment with either an SGLT2i or metformin without exposure to either drug (n = 182 160)	Prior PD, secondary parkinsonism, or anti-parkinsonian drug use	PS matching on age, sex, ethnicity, comorbidities, and comedications	Date of first prescription	≥1 year of continuous prescription	Metformin (n = 91 080); SGLT2i (n = 91 080)	PD diagnosis, mortality; IPTW analysis and Cox proportional hazard regression	SGLT2i: 987.44 (603.2); Metformin: 944.7 (591.5)† (in days)
Mui *et al.* (2021) [24]	Retrospective, territory-wide population-based cohort study from January 2015 to December 2019 (Hong Kong)	T2DM patients with SGLT2i/DPP4i use between January 2015 and December 2019 (n = 39 828)	Prior neurological/psychiatric disease	PS matching on CCI, comorbidities, non-SGLT2i/DPP4i medications, baseline fasting glucose, and HbA1c	Date of first prescription	≥1 month use of SGLT2i or DPP4i prescription	SGLT2i (n = 13 283); DPP4i (n = 26 545)	PD and AD diagnosis, mortality; Cox regression model	427† (in days)
Zhao *et al.* (2022) [30]	Longitudinal information of EMR from YRHCD (China)	Adult patients registered in the DM registry system and diagnosed with T2DM or had >2 diagnosis records of T2DM and no records of type 1 DM in the EMR (n = 62 400)	PD diagnosis before the index date	IPTW applied for age, sex, comedications, BMI, cholesterol panel, HbA1c, and medical use	First date of prescription fill	≥2 consecutive prescriptions within 6 months of the index date	AGI (n = 49 696); TZD (n = 12 704)	PD diagnosis; Cox regression model, subgroup analysis on cumulative duration, per-protocol sensitivity analysis	2.2†

AD – Alzheimer disease, AGI – alpha-glucosidase inhibitor, CCI – Charlson comorbidity index, DM – diabetes mellitus, DPP4i – dipeptidyl peptidase-4 inhibitor, EMR – electronic medical record, GLP-1RA – glucagon-like peptide-1 receptor agonist, HWDC – Health and Welfare Data Centre, IPTW – inverse probability of treatment weighting, NA – not available, PD – Parkinson disease, PS – propensity score, SGLT2i – sodium-glucose co-transporter-2 inhibitor, T2DM – type 2 diabetes mellitus, TZD – thiazolidinediones, YRHCD – Yinzhou Regional Health Care Database

*Values presented as median (interquartile range).

†Values presented as means (standard deviations).

We examined the distribution of key effect modifiers across studies to assess the transitivity assumption and observed generally comparable characteristics across treatment comparisons and supporting the transitivity assumption. The quality assessment showed the studies had a moderate level of bias. Both visual inspection of funnel plots and Egger’s test indicated a low likelihood of publication bias; however, this should be interpreted cautiously given the limited number of included studies. No significant inconsistency was detected between direct and indirect evidence based on node-splitting analyses (Table S2 and Figures S1–3 in the **Online Supplementary Document**).

### Incident PD risk across antidiabetic classes

**Figure 2:**
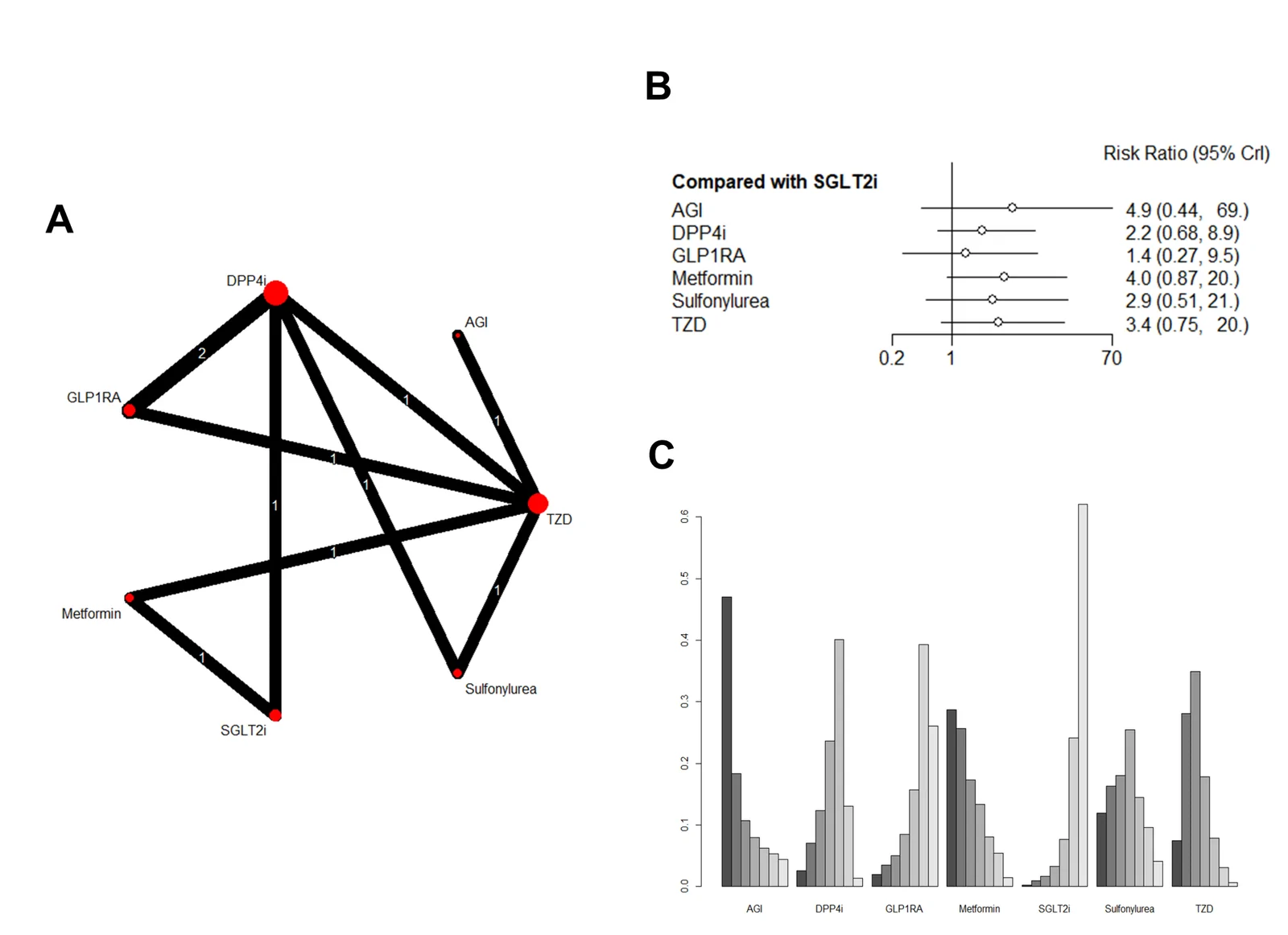
Incident Parkinson disease risk. **Panel A.** Network plot. **Panel B.** Risk of incident Parkinson disease associated with antidiabetics. **Panel C.** Rank of probability. AGI – α-glucosidase inhibitor, CrI – credible interval, DPP4i – dipeptidyl peptidase-4 inhibitor, GLP-1RA – glucagon-like peptide-1 receptor agonist, SGLT2i – sodium-glucose co-transporter-2 inhibitor, TZD – thiazolidinedione. Node size indicates the total sample size for each treatment, and line thickness represents the number of direct head-to-head comparisons in the network plot.

The most prevalent comparison pair in the network plot on PD risk across antidiabetic classes was DPP4i and SGLT2i (**Figure 2**, Panel A; **Table 2**). Compared with SGLT2 inhibitors, other antidiabetic classes demonstrated higher point estimates for PD risk; however, the wide CrIs overlapping the null indicated substantial uncertainty and lack of statistically significant differences (**Figure 2**, Panel B). The estimated between-study heterogeneity was moderate, with a posterior mean between-study standard deviation of 0.83 (95% CrI = 0.33–1.58). SGLT2 inhibitors had the highest probability of being associated with the lowest PD risk, followed by GLP-1RA and DPP4 inhibitors, whereas AGI and sulfonylureas ranked lower, with wide distributions reflecting uncertainty. (**Figure 2**, Panel C). The overall SUCRA rankings indicated that SGLT2 inhibitors had the highest SUCRA value (Table S3 in the **Online Supplementary Document**).

**Table 2:** League table of network meta-analysis*

AGI	0.44 (0.05–4.68)	0.29 (0.03–3.61)	0.813 (0.06–9.48)	0.20 (0.01–2.26)	0.58 (0.05–6.80)	0.70 (0.11–4.53)
2.25 (0.21–21.55)	**DPP4i**	0.65 (0.17–2.42)	1.83 (0.32–9.13)	0.453 (0.11–1.47)	1.31 (0.29–6.00)	1.57 (0.40–5.68)
3.43 (0.28–39.01)	1.53 (0.41–5.76)	**GLP-1RA**	2.80 (0.35–19.31)	0.69 (0.11–3.70)	2.01 (0.31–13.23)	2.42 (0.49–11.63)
1.23 (0.11–15.48)	0.55 (0.11–3.10)	0.36 (0.05–2.83)	**Metformin**	0.25 (0.05–1.14)	0.72 (0.10–5.77)	0.86 (0.19–4.29)
4.94 (0.44–68.64)	2.21 (0.68–8.91)	1.44 (0.27–9.47)	4.03 (0.87–20.38)	**SGLT2i**	2.89 (0.51–21.05)	3.43 (0.75–19.64)
1.71 (0.15–18.84)	0.76 (0.17–3.42)	0.50 (0.08–3.26)	1.39 (0.17–9.67)	0.35 (0.05–1.98)	**Sulfonylurea**	1.21 (0.26–5.22)
1.43 (0.22–9.45)	0.64 (0.18–2.48)	0.41 (0.09–2.05)	1.17 (0.23–5.32)	0.29 (0.05–1.33)	0.83 (0.19–3.80)	**TZD**

AGI – α-glucosidase inhibitor, DPP4i – dipeptidyl-peptidase-4 inhibitor, GLP-1RA – glucagon-like peptide-1 receptor agonist, SGLT2i – sodium-glucose co-transporter-2 inhibitor, TZD – thiazolidinediones

*Values are presented as risk ratios (95% credible intervals).

### Subgroup analysis

**Figure 3:**
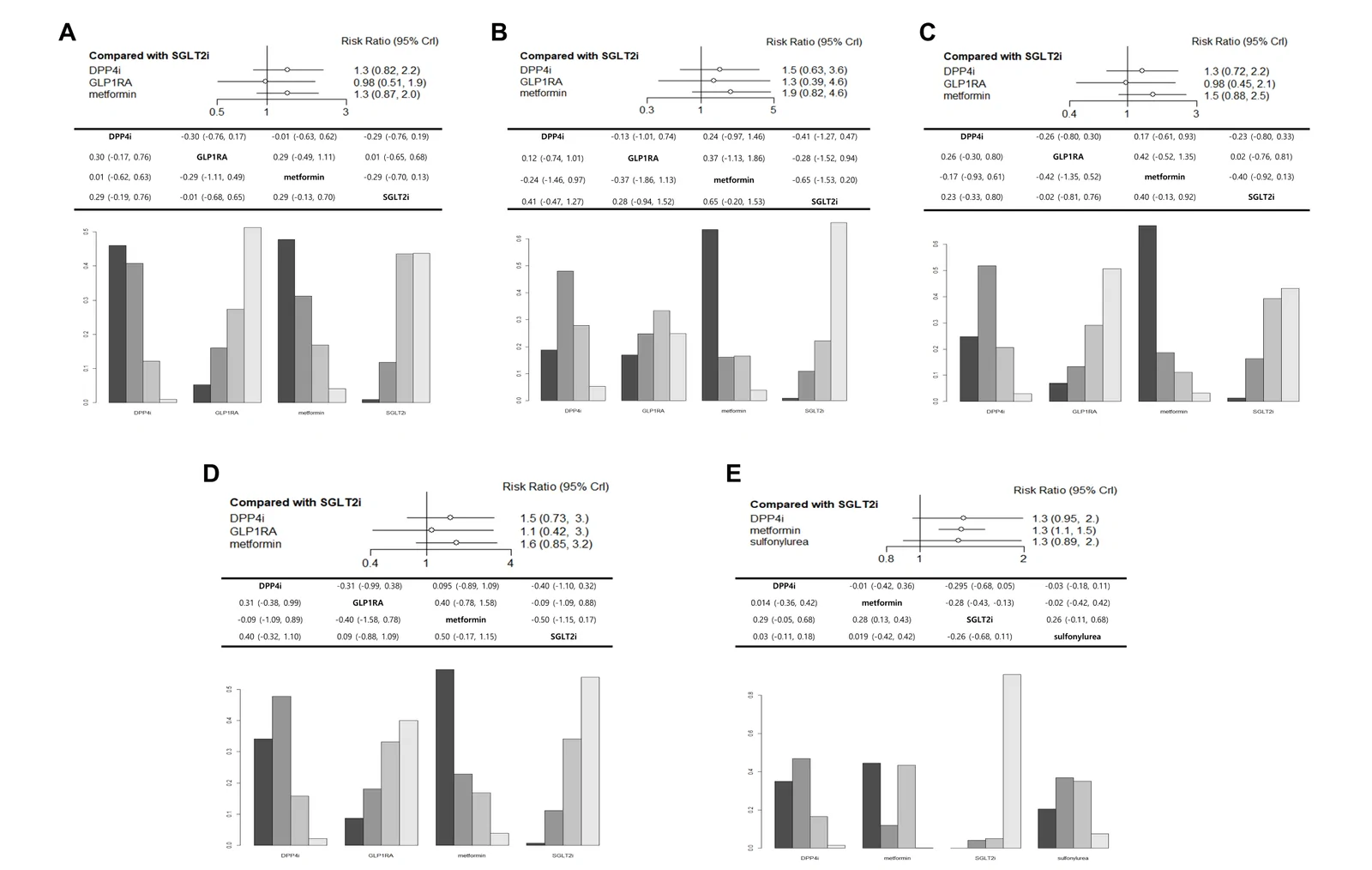
Subgroup analysis. **Panel A.** Age <75 years. **Panel B.** Age ≥75 years. **Panel C.** Men. **Panel D.** Women. **Panel E.** CVD history. CVD – cardiovascular disease, CrI – credible interval, DPP4i – dipeptidyl peptidase-4 inhibitor, GLP-1RA – glucagon-like peptide-1 receptor agonist, SGLT2i – sodium-glucose co-transporter-2 inhibitor.

We conducted subgroup analyses of the association between specific antidiabetic classes and PD risk (**Figure 3**, Panels A–E). The probability ranking suggested that DPP4i was more likely to be associated with increased PD risk in patients <75 years. Among patients aged ≥75 years, point estimates indicated relatively higher PD risk; however, uncertainty remained due to wide CrIs. No significant differences in PD risk were observed across antidiabetic classes in sex-stratified subgroup analysis. In patients with CVD comorbidities, the risk of PD was higher with metformin than with SGLT2i (RR = 1.30; 95% CrI = 1.10–1.50), while sulfonylureas and DPP4is showed comparable risks. Rank probability analysis further suggested that metformin was consistently associated with a higher rank for PD risk across age-, sex- and comorbidity-stratified groups. In contrast, SGLT2i was associated with the lowest PD risk among older patients (≥75 years), women, and those with previous CVD history, whereas GLP-1RA demonstrated a higher probability of lower PD risk in men and individuals <75 years.

In sensitivity analyses restricted to studies with a follow-up duration of ≥2 years, the overall pattern of associations between antidiabetic drug classes and PD risk remained consistent with the primary analysis (Table S4 in the **Online Supplementary Document**). When compared with SGLT2i, no antidiabetic drug class demonstrated a statistically meaningful difference in PD risk, as all 95% CrIs crossed the null value (Figure S4 in the **Online Supplementary Document**). The SUCRA rankings from the sensitivity analyses indicated that GLP-1RA had the highest SUCRA value (Table S3 in the **Online Supplementary Document**). Sulfonylureas consistently ranked lowest across both overall and sensitivity analyses.

## DISCUSSION

We found that metformin and AGI were associated with higher point estimates for PD risk, whereas DPP4i, GLP-1RA, SGLT2i, and TZD generally demonstrated lower values. However, most comparisons were not statistically significant. Rank probability analyses suggested that GLP-1RA and SGLT2i were associated with lower PD risk across age-, sex-, and comorbidity-stratified analyses. Subgroup analyses further indicated that GLP-1RA had a higher probability of lower PD risk in individuals <75 years and in men, whereas SGLT2i tended to rank lower in PD risk among older patients (≥75 years), women, and those with a history of CVD. Yet findings should be interpreted cautiously given the limited number of studies and wide CrIs.

Diabetes-related pathophysiological changes, including chronic hyperglycaemia, insulin resistance, mitochondrial dysfunction, and neuroinflammation, are recognised contributors to neurodegeneration [32]. The American Diabetes Association guidelines recommend careful consideration of comorbid conditions, including cognitive impairment, when selecting antidiabetic therapy, especially in older populations, emphasising the importance of minimising hypoglycaemia risk [21]. However, evidence regarding the effect of hypoglycaemia on PD risk remains limited [21]. We could not fully determine the direct impact of hypoglycaemia on PD risk. Neither AGI nor sulfonylurea demonstrated a statistically significant increase in PD risk compared with SGLT2i, and the estimates remained imprecise with wide CrIs. However, AGIs consistently ranked higher in PD risk across SUCRA-based ranking analyses. Moreover, frequentist pairwise comparisons showed that several drug classes, including DPP4i (RR = 0.25; 95% confidence interval (CI) = 0.07–0.82), GLP-1RA (RR = 0.19; 95% CI = 0.05–0.70), and SGLT2i (RR = 0.15; 95% CI = 0.04–0.55), were associated with lower PD risk compared with AGI (Figure S5 in the **Online Supplementary Document**). These findings suggest that AGI may be associated with a less favourable neurological risk profile, although caution is warranted due to the limited number of studies in our analysis and potential residual confounding. Importantly, patients with PD frequently experience mild cognitive impairment, which often progresses to PD-related dementia, implying that this population may be particularly vulnerable to hypoglycaemia-induced neuronal injury [33]. Therefore, further longitudinal studies with extended follow-up and systematic capture of hypoglycaemia incidence across antidiabetic regimens are warranted to clarify whether differences in PD risk are mediated by hypoglycaemia and whether minimising hypoglycaemia incidence may reduce PD risk or slow disease progression.

Our findings suggest that SGLT2i and GLP-1RA were associated with lower PD risk, particularly among older adults and patients with CVD. However, we urge caution when interpreting our results, as our analysis covered only nine studies. Yet these findings are still clinically meaningful, considering the well-established risk factors of PD, which include advanced age, male sex, genetic susceptibility, and environmental exposure to chemicals (*e.g.* pesticides) [34]. In addition, metabolic disorders such as diabetes have been recognised as major contributors to neurodegeneration, whereby systemic metabolic dysregulation accelerates neuronal vulnerability [35]. Recent evidence also suggests that CVD may serve as a risk factor for the development and progression of PD [36]. Accumulating evidence further indicates that the risk of CVD, including myocardial infarction, stroke, and heart failure, is substantially elevated in patients with PD and is strongly correlated with disease severity [37,38]. The observed association between diabetes, CVD, and PD may be partly explained by shared pathophysiological mechanisms. Autonomic dysfunction in PD leads to blood pressure instability and arrhythmias, while diabetes and chronic hyperglycaemia further exacerbate cardiovascular vulnerability, reinforcing the interconnected pathways linking metabolic disease, cardiovascular health, and neurodegeneration [39,40]. Therefore, the observed association with lower PD risk with SGLT2i and GLP-1RA may partly reflect their favourable cardiometabolic profiles rather than direct neuroprotective effects. SGLT2i has been shown to reduce heart failure and major adverse cardiovascular events, potentially alleviating downstream neurodegenerative burden [18]. GLP-1RAs may exert neuroprotection through anti-inflammatory and mitochondrial effects, and an RCT is currently underway to evaluate the effect of exenatide on sustained motor outcomes in PD patients [41,42].

The subgroup findings suggest that SGLT2i consistently rank as having lower PD risk among older patients, women and those with CVD, whereas GLP-1RA demonstrated a higher probability of lower PD risk in men and individuals aged <75 years. These patterns may partly reflect differences in the cardiometabolic effects of these agents. In older patients, who are more likely to have renal impairment due to long-standing diabetes, the cardiorenal protective effects of SGLT2i may translate into additional neuroprotective benefits, as implied by evidence showing a significant association between renal insufficiency and increased PD risk [43]. In contrast, GLP-1RA may exert greater protective effects in men, among whom metabolic-associated steatohepatitis, metabolic inflammation, and metabolic syndromes are more prevalent, which may be related to gender differences in metabolic inflammation, although further verification is required [44,45]. Nevertheless, these subgroup findings should be interpreted cautiously, and further large-scale, controlled studies should validate them and elucidate the underlying mechanisms.

While we found a comparatively higher probability of PD risk with metformin, AGIs, and sulfonylurea, we cannot explain what the mechanisms underlie these associations. Although long-term metformin use has been associated with vitamin B12 deficiency, which may contribute to neuropathy, cognitive decline, and increased vulnerability to neurodegeneration [46], prior studies have also reported neutral or potentially neuroprotective effects of metformin in neurodegenerative disorders [13]. Hence, the association identified in our analysis should be interpreted with care. Clinically, metformin is usually prescribed at earlier stages of diabetes, and thus users may differ systematically in baseline neurological risk compared with patients receiving later-line therapies [18,32]. Furthermore, recent guidelines no longer designate metformin as mandatory first-line therapy, instead emphasising individualised treatment selection based on comorbidities such as cardiovascular and renal disease [21]. Lastly, metformin is frequently used as background therapy in patients receiving combination regimens, which may reflect differences in diabetes duration, disease severity, and comorbidity burden. These prescribing patterns raise the possibility of confounding by indication and reverse causation, particularly when treatment groups differ in baseline metabolic and neurological risk profiles. Therefore, the observed associations may reflect underlying patient characteristics rather than direct pharmacologic effects, and further large-scale prospective studies should clarify the relationship between metformin use and PD risk.

To the best of our knowledge, this study is the first to evaluate PD risk across multiple antidiabetic classes using stratified patient characteristics. However, several limitations should be acknowledged in relation to its findings. First, as all included studies were observational in nature, residual confounding cannot be fully excluded, despite adjustment for measured covariates using multivariable regression or propensity score-based methods. Potential confounding by indication and reverse causation should also be considered. Patients receiving newer agents may differ systematically from those receiving older drugs in clinical characteristics that influence PD risk, and early prodromal symptoms of PD may affect treatment selection. Although several studies used propensity-score adjustment and lag-time analyses, residual confounding cannot be excluded. Moreover, heterogeneity may have been introduced due to variations in study design, exposure definitions, inclusion and exclusion criteria, and follow-up durations across studies. Although most studies defined the index date as the first prescription and exposure as ≥2 consecutive prescriptions, minor variations may have influenced comparability across studies.

Furthermore, while we excluded studies comparing drug users *vs.* non-users within the same class and case-control studies to improve comparability and internal validity, this approach may have reduced the overall evidence base and statistical power of the network. Additionally, the relatively small number of studies and sparse treatment comparisons within the network may have increased reliance on indirect evidence, potentially reducing the stability of effect estimates and the robustness of treatment rankings and CrIs. Subgroup analyses were also based on limited study-level data, which may have reduced statistical power. Moderate between-study heterogeneity and the absence of global inconsistency assessment using a design-by-treatment interaction model may have further contributed to uncertainty. Furthermore, the inability to fully account for important therapeutic factors, such as treatment duration, dose of antidiabetic therapy, types of concomitant medications, severity of diabetes, and glycaemic control status, may have influenced the observed associations. Data on two major contributors for PD, disease duration and haemoglobin A1C levels, were rarely reported, limiting the ability to adjust for these factors. Most included cohorts originated in East Asia or the USA, which may limit generalisability. Furthermore, the restriction of English-language publications may have introduced language bias. Additionally, publication bias assessment may have been underpowered given the small number of studies. Lastly, information on factors accelerating neurodegeneration, such as hypoglycaemia incidence, vitamin B12 levels, and other metabolic indicators, was also limited.

Despite these limitations, this study is the most comprehensive synthesis of PD risk across antidiabetic drug classes, encompassing more than 711 000 patients from diverse geographic regions, with subgroup analyses by age, sex, and CVD history providing clinically relevant insights. Nevertheless, further studies are warranted to clarify the causal relationship between antidiabetic therapy and PD risk, to determine sex- and age-specific responses, and to explore the modifying effect of comorbidities such as CVD and chronic kidney disease.

## CONCLUSIONS

Our findings suggest that SGLT2i and GLP-1RA are associated with lower PD risk, while metformin, AGIs and sulfonylureas demonstrated comparatively less favourable ranking probabilities, with no statistically significant differences across most comparisons. Subgroup analyses on age-, sex-, and comorbidity-specific differences suggested potential heterogeneity in treatment effects, emphasising the importance of individualised therapeutic approaches. These results emphasise that the effect of antidiabetic agents may extend beyond glycaemic control, potentially mitigating neurodegenerative risk. They further suggest that the selection of antidiabetic therapies should consider long-term neurological benefits in addition to metabolic and cardiovascular outcomes, with particular attention to hypoglycaemic risk in patients at elevated PD risk. Further controlled studies are warranted to validate these associations and to determine whether antidiabetic therapies can influence PD onset, progression, or long-term neurological outcomes.

## Additional material


Online Supplementary Document

## Data Availability

**Data availability:** All data generated or analysed during this study are included in the cited publications and their online supplementary files, as well as in our own article and its **Online Supplementary Document**

## References

[1] Poewe W Seppi K Tanner CM Halliday GM Brundin P Volkmann J Parkinson disease. Nat Rev Dis Primers. 2017;3:17013. 10.1038/nrdp.2017.13 28332488

[2] Ben-Shlomo Y Darweesh S Llibre-Guerra J Marras C San Luciano M Tanner C The epidemiology of Parkinson’s disease. Lancet. 2024;403:283–92. 10.1016/S0140-6736(23)01419-8 38245248 PMC11123577

[3] Su D Cui Y He C Yin P Bai R Zhu J Projections for prevalence of Parkinson’s disease and its driving factors in 195 countries and territories to 2050: modelling study of Global Burden of Disease Study 2021. BMJ. 2025;388:e080952. 10.1136/bmj-2024-080952 40044233 PMC11881235

[4] Burchill E Watson CJ Fanshawe JB Badenoch JB Rengasamy E Ghanem DA The impact of psychiatric comorbidity on Parkinson’s disease outcomes: a systematic review and meta-analysis. Lancet Reg Health Eur. 2024;39:100870. 10.1016/j.lanepe.2024.100870 38361749 PMC10867667

[5] Pringsheim T Day GS Smith DB Rae-Grant A Licking N Armstrong MJ Dopaminergic therapy for motor symptoms in early Parkinson disease practice guideline summary: A report of the AAN guideline subcommittee. Neurology. 2021;97:942–57. 10.1212/WNL.0000000000012868 34782410 PMC8672433

[6] Bantounou MA Shoaib K Mazzoleni A Modalavalasa H Kumar N Philip S The association between type 2 diabetes mellitus and Parkinson’s disease; a systematic review and meta-analysis. Brain Disord. 2024;15:100158. 10.1016/j.dscb.2024.100158

[7] König A Outeiro TF Diabetes and Parkinson’s disease: Understanding shared molecular mechanisms. J Parkinsons Dis. 2024;14:917–24. 10.3233/JPD-230104 38995799 PMC11307096

[8] Yan MH Wang X Zhu X Mitochondrial defects and oxidative stress in Alzheimer disease and Parkinson disease. Free Radic Biol Med. 2013;62:90–101. 10.1016/j.freeradbiomed.2012.11.014 23200807 PMC3744189

[9] Béraud D Maguire-Zeiss KA Misfolded α-synuclein and Toll-like receptors: therapeutic targets for Parkinson’s disease. Parkinsonism Relat Disord. 2012;18:S17–20. 10.1016/S1353-8020(11)70008-6 22166424 PMC3500631

[10] Ransohoff RM How neuroinflammation contributes to neurodegeneration. Science. 2016;353:777–83. 10.1126/science.aag2590 27540165

[11] Fiory F Perruolo G Cimmino I Cabaro S Pignalosa FC Miele C The relevance of insulin action in the dopaminergic system. Front Neurosci. 2019;13:868. 10.3389/fnins.2019.00868 31474827 PMC6706784

[12] Sunwoo Y Park J Choi CY Shin S Choi YJ Risk of dementia and Alzheimer’s disease associated with antidiabetics: A Bayesian network meta-analysis. Am J Prev Med. 2024;67:434–43. 10.1016/j.amepre.2024.04.014 38705542

[13] Hu L Wang W Chen X Bai G Ma L Yang X Prospects of antidiabetic drugs in the treatment of neurodegernative disease. Brain-X. 2024;2:e52. 10.1002/brx2.52

[14] Albuquerque MB Nunes L Oliveira Maldonado JV Melo Ferreira DG Margato MM Rabelo LV GLP-1 receptor agonists for Parkinson’s disease: An updated meta-analysis. Parkinsonism Relat Disord. 2025;130:107220. 10.1016/j.parkreldis.2024.107220 39642803

[15] Hutton B Salanti G Caldwell DM Chaimani A Schmid CH Cameron C The PRISMA extension statement for reporting of systematic reviews incorporating network meta-analyses of health care interventions: checklist and explanations. Ann Intern Med. 2015;162:777–84. 10.7326/M14-2385 26030634

[16] Sterne JA Hernán MA Reeves BC Savović J Berkman ND Viswanathan M ROBINS-I: a tool for assessing risk of bias in non-randomised studies of interventions. BMJ. 2016;355:i4919. 10.1136/bmj.i4919 27733354 PMC5062054

[17] Cochrane. Chapter 11: Undertaking network meta-analyses. 2019. Available: https://www.cochrane.org/authors/handbooks-and-manuals/handbook/current/chapter-11. Accessed 5 August 2025.

[18] Forzano I Wilson S Lombardi A Jankauskas SS Kansakar U Mone P SGLT2 inhibitors: an evidence-based update on cardiovascular implications. Expert Opin Investig Drugs. 2023;32:839–47. 10.1080/13543784.2023.2263354 37740906 PMC10591907

[19] Zelniker TA Braunwald E Mechanisms of cardiorenal effects of sodium-glucose cotransporter 2 inhibitors: JACC State-of-the-Art Review. J Am Coll Cardiol. 2020;75:422–34. 10.1016/j.jacc.2019.11.031 32000955

[20] Kim HK Biessels GJ Yu MH Hong N Lee YH Lee BW SGLT2 inhibitor use and risk of dementia and Parkinson disease among patients with type 2 diabetes. Neurology. 2024;103:e209805. 10.1212/WNL.0000000000209805 39292986

[21] American Diabetes Association Professional Practice Committee Summary of revisions: Standards of care in diabetes-2025. Diabetes Care. 2025;48:S6–13. 10.2337/dc25-SREV 39651984 PMC11635056

[22] Lin L Chu H Quantifying publication bias in meta-analysis. Biometrics. 2018;74:785–94. 10.1111/biom.12817 29141096 PMC5953768

[23] Brakedal B Flønes I Reiter SF Torkildsen Ø Dölle C Assmus J Glitazone use associated with reduced risk of Parkinson’s disease. Mov Disord. 2017;32:1594–9. 10.1002/mds.27128 28861893 PMC5697685

[24] Mui JV Zhou J Lee S Leung KSK Lee TTL Chou OHI Sodium-glucose cotransporter 2 (SGLT2) inhibitors vs. dipeptidyl peptidase-4 (DPP4) inhibitors for new-onset dementia: A propensity score-matched population-based study with competing risk analysis. Front Cardiovasc Med. 2021;8:747620. 10.3389/fcvm.2021.747620 34746262 PMC8566991

[25] Sun M Wang X Lu Z Yang Y Lv S Miao M SGLT2 inhibitors vs. metformin for Parkinson’s disease risk reduction in type 2 diabetes. J Parkinsons Dis. 2025;15:1240–52. 10.1177/1877718X251359391 40671477 PMC13347531

[26] Guo J Tang H Shao H Lu Y Shi L Fonseca VA Sodium-glucose cotransporter 2 inhibitors and the risk of Parkinson disease in real-world patients with type 2 diabetes. Diabetes Obes Metab. 2024;26:5727–36. 10.1111/dom.15943 39256938 PMC12135020

[27] Tang H Lu Y Okun MS Donahoo WT Ramirez-Zamora A Wang F Glucagon-like peptide-1 receptor agonists and risk of Parkinson’s disease in patients with type 2 diabetes: A population-based cohort study. Mov Disord. 2024;39:1960–70. 10.1002/mds.29992 39189078 PMC11568939

[28] Connolly JG Bykov K Gagne JJ Thiazolidinediones and Parkinson disease: A cohort study. Am J Epidemiol. 2015;182:936–44. 10.1093/aje/kwv109 26493264 PMC4655743

[29] Huang KH Yang Y Gau SY Tsai TH Lee CY Association between dipeptidyl peptidase-4 inhibitor use and risk of Parkinson’s disease among patients with diabetes mellitus: a retrospective cohort study. Aging (Albany NY). 2024;16:11994–2007. 10.18632/aging.206074 39177655 PMC11386917

[30] Zhao H Zhuo L Sun Y Shen P Lin H Zhan S Thiazolidinedione use and risk of Parkinson’s disease in patients with type 2 diabetes mellitus. NPJ Parkinsons Dis. 2022;8:138. 10.1038/s41531-022-00406-8 36271052 PMC9587207

[31] Brauer R Wei L Ma T Athauda D Girges C Vijiaratnam N Diabetes medications and risk of Parkinson’s disease: a cohort study of patients with diabetes. Brain. 2020;143:3067–76. 10.1093/brain/awaa262 33011770 PMC7794498

[32] Baduini IR Castro Vildosola JE Kavehmoghaddam S Kiliç F Nadeem SA Nizama JJ Type 2 diabetes mellitus and neurodegenerative disorders: The mitochondrial connection. Pharmacol Res. 2024;209:107439. 10.1016/j.phrs.2024.107439 39357690

[33] Aarsland D Batzu L Halliday GM Geurtsen GJ Ballard C Ray Chaudhuri K Parkinson disease-associated cognitive impairment. Nat Rev Dis Primers. 2021;7:47. 10.1038/s41572-021-00280-3 34210995

[34] Beheshti I Exploring risk and protective factors in Parkinson’s disease. Cells. 2025;14:710. 10.3390/cells14100710 40422213 PMC12110598

[35] Grotewold N Albin RL Update: Protective and risk factors for Parkinson disease. Parkinsonism Relat Disord. 2024;125:107026. 10.1016/j.parkreldis.2024.107026 38879999 PMC11846500

[36] Acharya S Lumley AI Devaux Y Cardiovascular history and risk of idiopathic Parkinson’s disease: a cross-sectional observational study. BMC Neurosci. 2024;25:33. 10.1186/s12868-024-00875-y 38977971 PMC11232247

[37] Park JH Kim DH Park YG Kwon DY Choi M Jung JH Association of Parkinson disease with risk of cardiovascular disease and all-cause mortality: A nationwide, population-based cohort study. Circulation. 2020;141:1205–7. 10.1161/CIRCULATIONAHA.119.044948 32250706

[38] Lim S Yum YJ Kim JH Lee CN Joo HJ Kwon DY Cardiovascular outcomes in Parkinson’s disease patients from a retrospective cohort study. Sci Rep. 2024;14:21928. 10.1038/s41598-024-72549-y 39304675 PMC11415384

[39] Kim JS Lee SH Oh YS Park JW An JY Park SK Cardiovascular autonomic dysfunction in mild and advanced Parkinson’s disease. J Mov Disord. 2016;9:97–103. 10.14802/jmd.16001 27020456 PMC4886202

[40] Pfeiffer RF Autonomic Dysfunction in Parkinson’s Disease. Neurotherapeutics. 2020;17:1464–79. 10.1007/s13311-020-00897-4 32789741 PMC7851208

[41] Siddeeque N Hussein MH Abdelmaksoud A Bishop J Attia AS Elshazli RM Neuroprotective effects of GLP-1 receptor agonists in neurodegenerative disorders: A large-scale propensity-matched cohort study. Int Immunopharmacol. 2024;143:113537. 10.1016/j.intimp.2024.113537 39486172

[42] Vijiaratnam N Girges C Auld G Chau M Maclagan K King A Exenatide once weekly over 2 years as a potential disease-modifying treatment for Parkinson’s disease: protocol for a multicentre, randomised, double blind, parallel group, placebo controlled, phase 3 trial: The 'Exenatide-PD3′ study. BMJ Open. 2021;11:e047993. 10.1136/bmjopen-2020-047993 34049922 PMC8166598

[43] Peng H Wu L Chen Q Chen S Wu S Shi X Association between kidney function and Parkinson’s disease risk: a prospective study from the UK Biobank. BMC Public Health. 2024;24:2225. 10.1186/s12889-024-19709-x 39148063 PMC11328353

[44] Joo SK Kim W Sex differences in metabolic dysfunction-associated steatotic liver disease: a narrative review. Ewha Med J. 2024;47:e17. 10.12771/emj.2024.e17 40703684 PMC12093579

[45] Zhong Y Wang TH Huang LJ Hua YS Association between metabolic syndrome and the risk of Parkinson’s disease: a meta-analysis. BMC Neurol. 2024;24:313. 10.1186/s12883-024-03820-y 39232681 PMC11373127

[46] Moore E Mander A Ames D Carne R Sanders K Watters D Cognitive impairment and vitamin B12: a review. Int Psychogeriatr. 2012;24:541–56. 10.1017/S1041610211002511 22221769

