# Supplementary material for: Comparative neuroprotective effects of antidiabetic medications on Parkinson's disease risk: a Bayesian network meta-analysis
Source: J Glob Health. 2026 Aug 21;16:04187. doi: 10.7189/jogh.16.04187 (PMC13494846; doi:10.7189/jogh.16.04187)
Supplement: Supplementary file 1 [file jogh-16-04187-s001.pdf]

Supplement to: Lee SH, Rho Y, Lee S, Shin S, Choi YJ. Comparative neuroprotective effects of antidiabetic medications on Parkinson’s disease risk: a Bayesian network meta-analysis. J Glob Health. 2026;16:04187.

Figure S1. Risk of bias

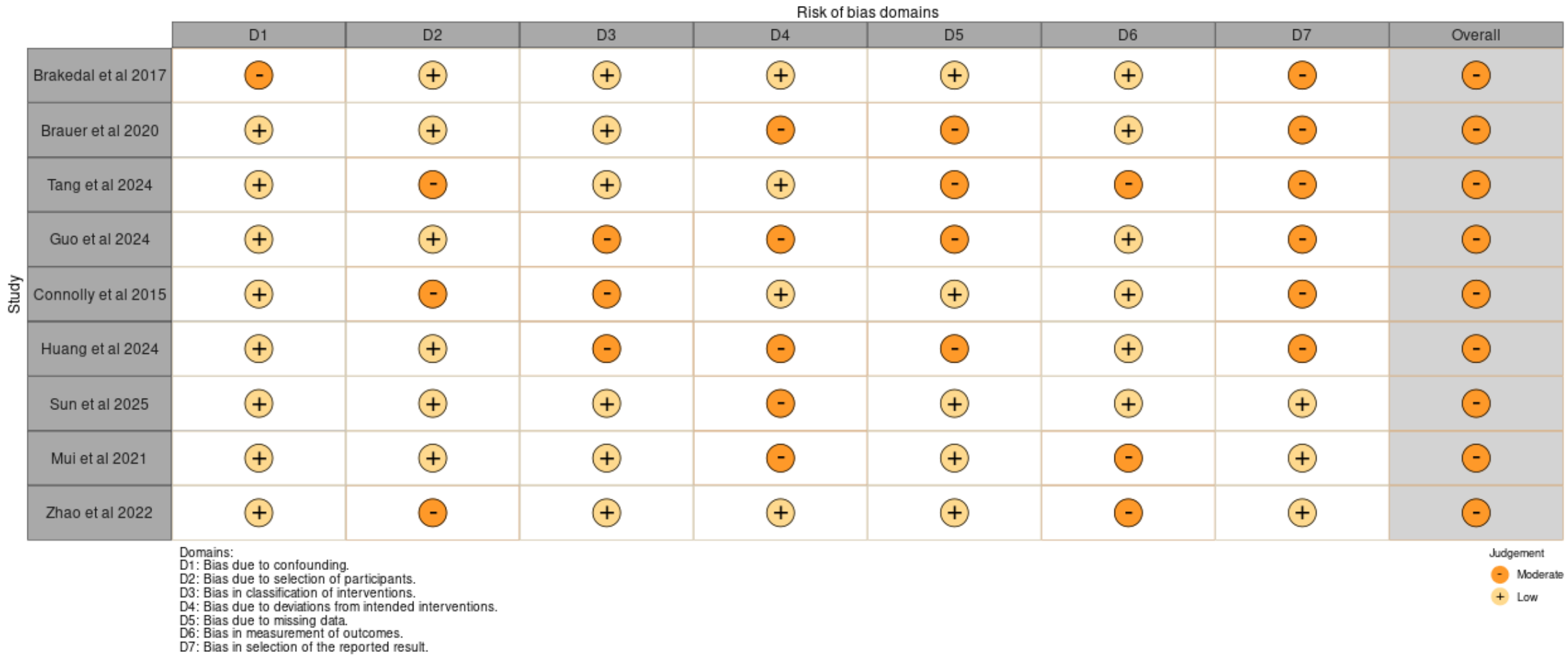

Figure S2. Publication bias

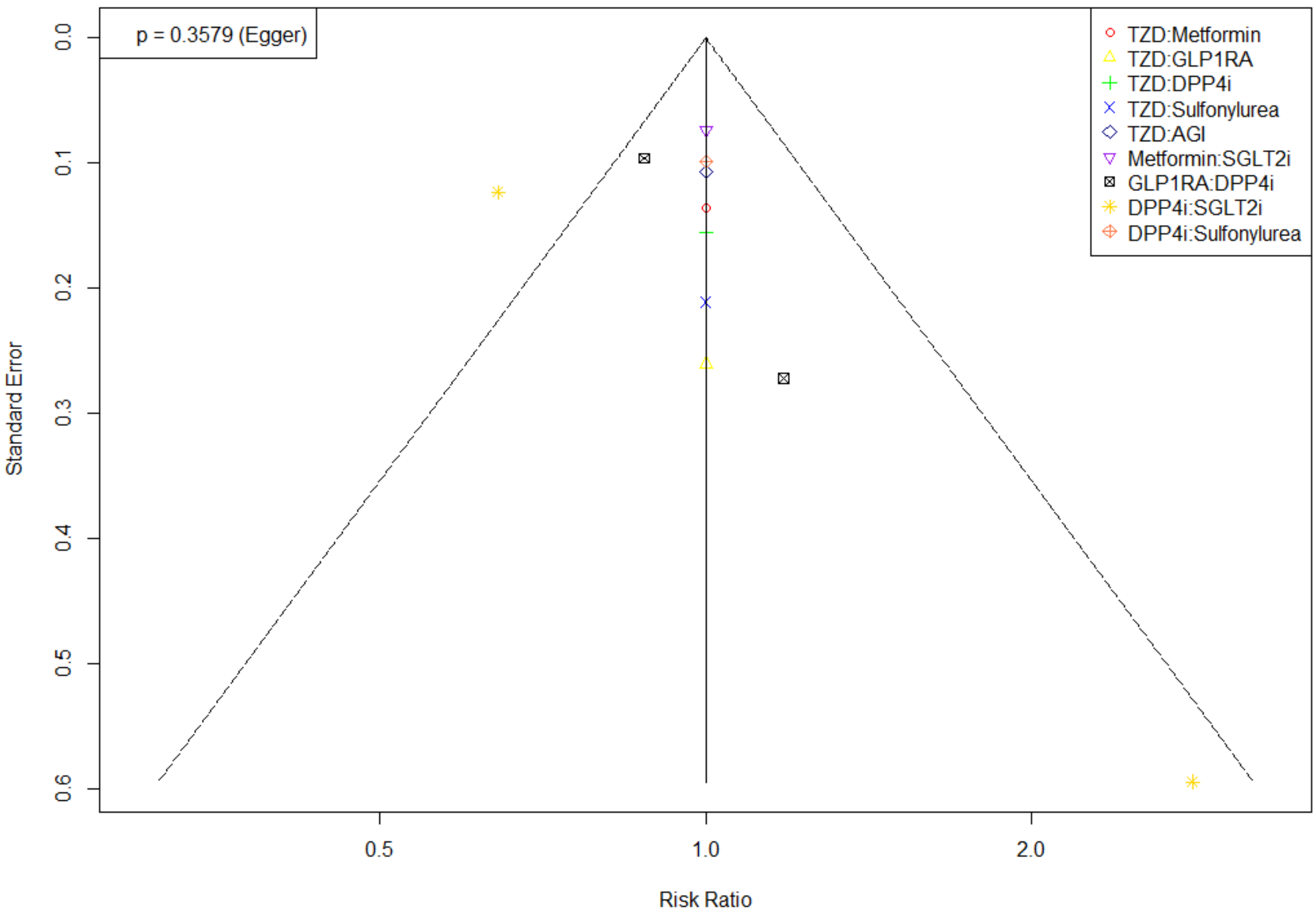

Figure S3. Node-split analysis

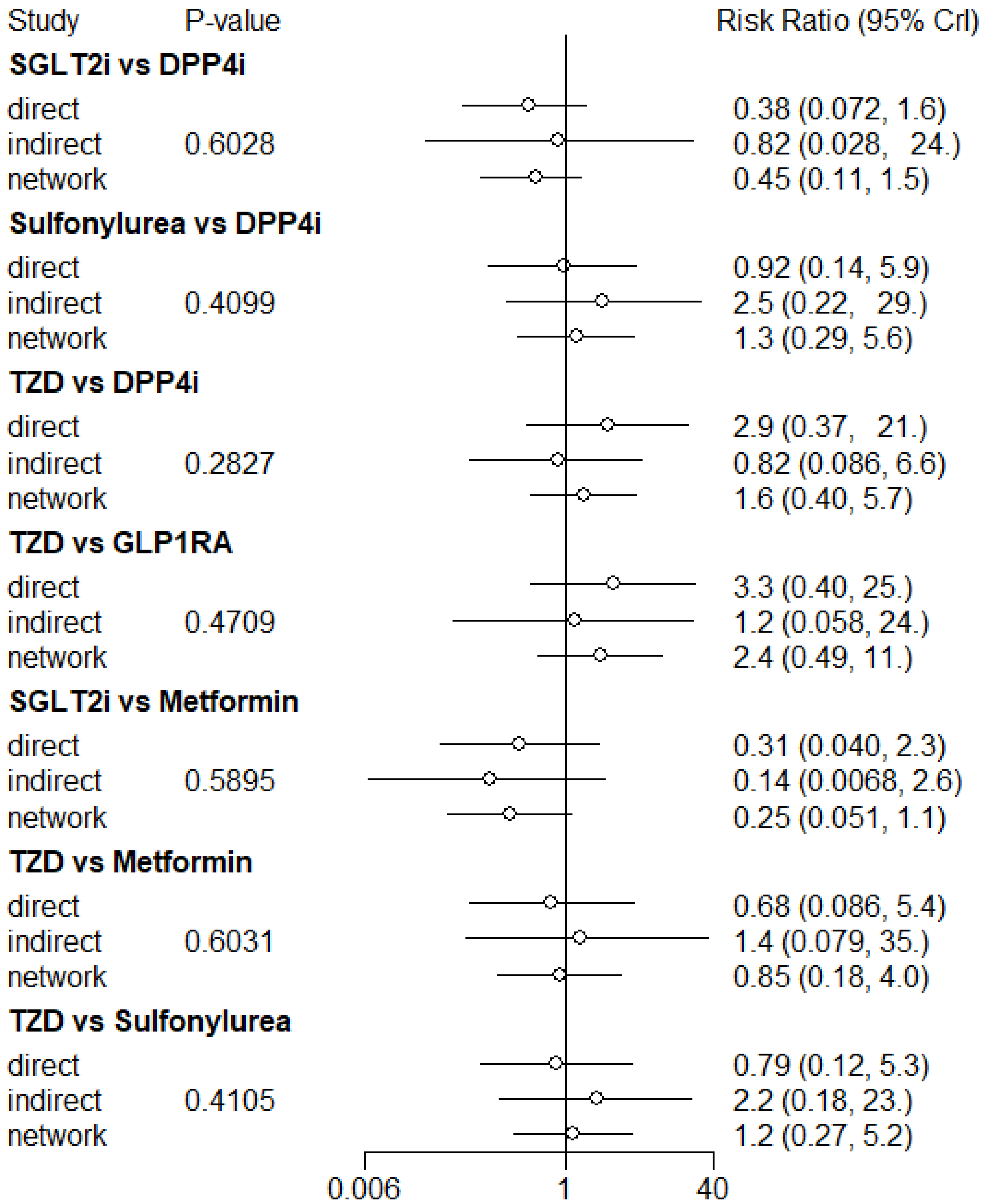

Figure S4. Sensitivity analysis. A) network plot, B) forest plot on PD risk

A.

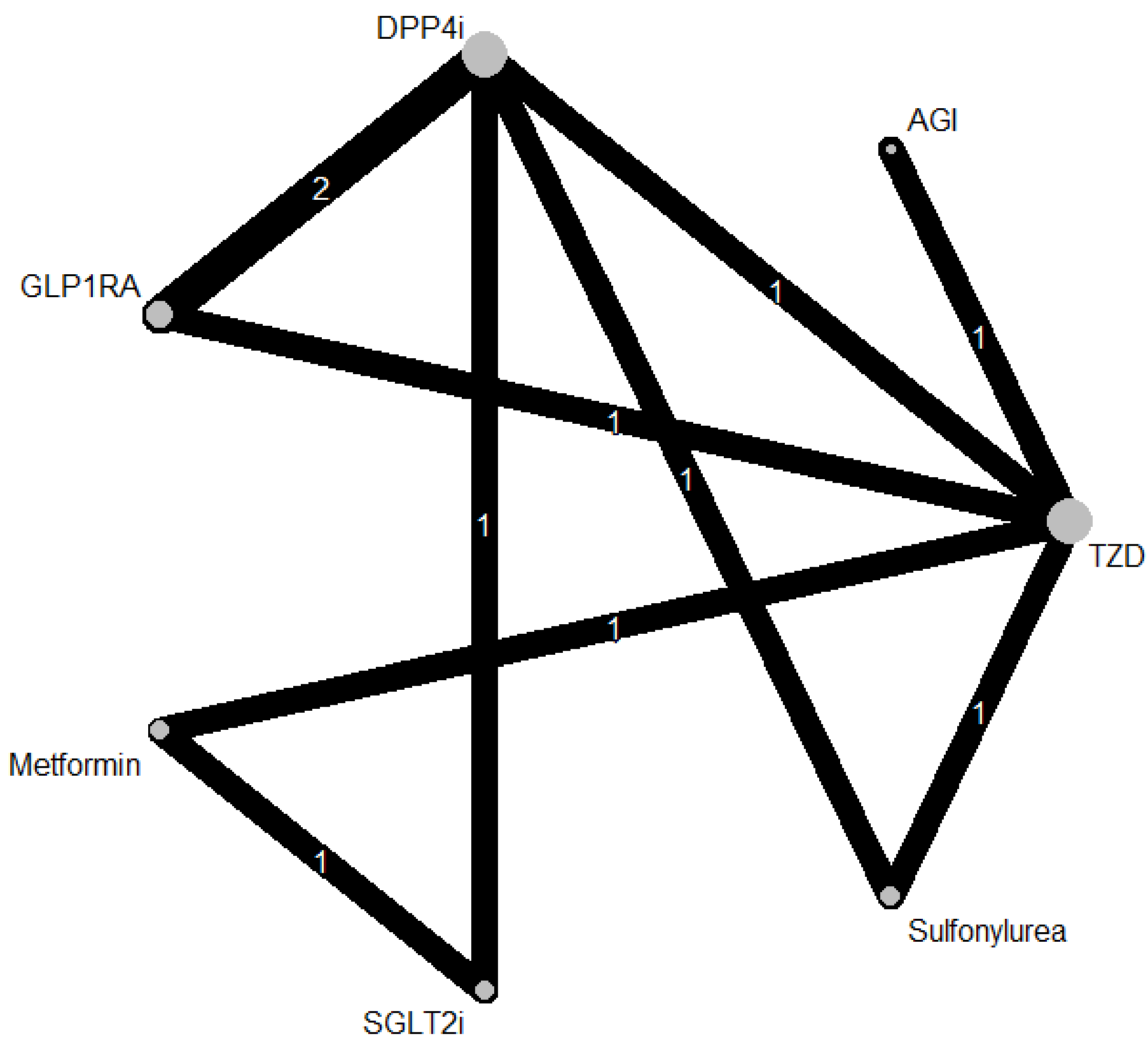

B.

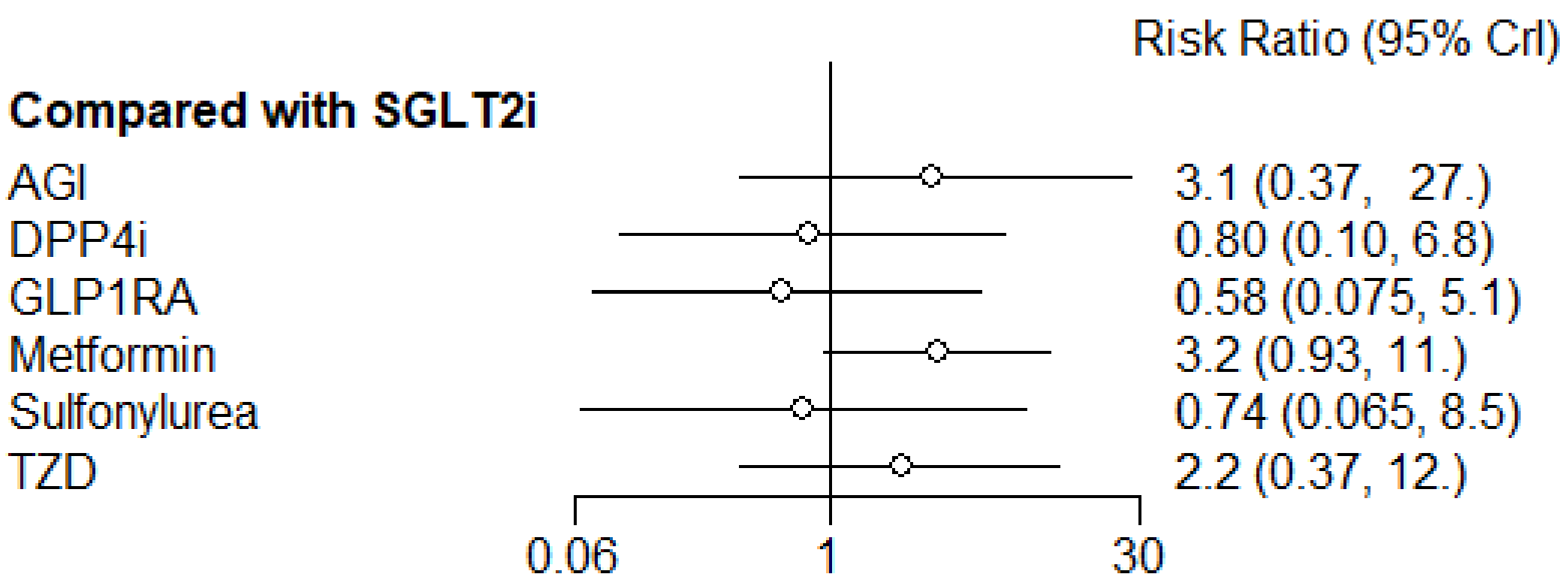

Figure S5. Frequentist analysis of PD risk across antidiabetic drug classes

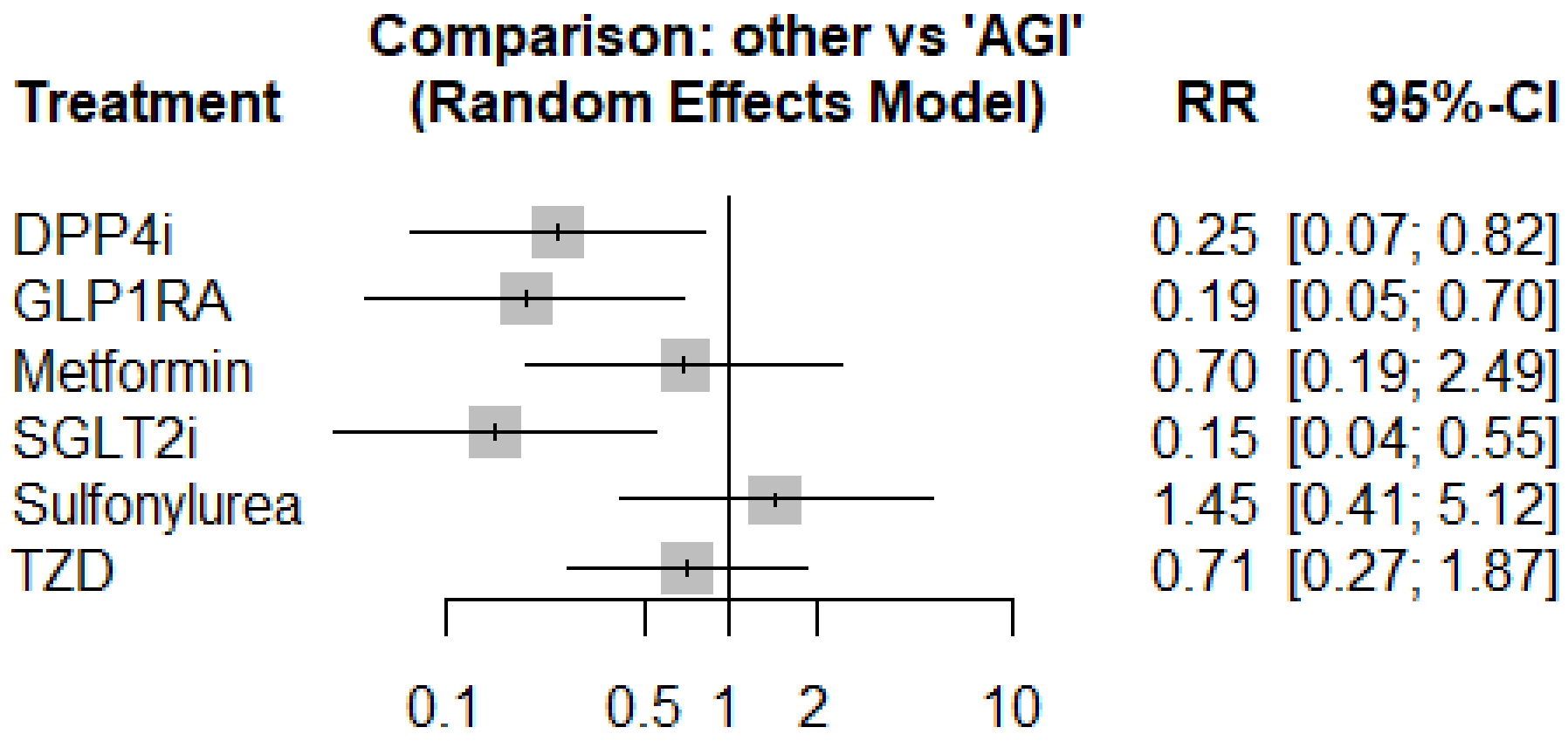

Table S1. Search strategy

Search duration	~ August 2025
Search Term	Number of studies
**PubMed**	
**#1:** 'Diabetes mellitus'[Mesh] OR diabetes Filters: Humans, exclude preprints	776,618
**#2:** parkinson disease Filters: Humans, excluded preprints	115,839
**#3:** "Hypoglycemic Agents"[All Fields] OR "antidiabetic" OR "hypoglycemic" OR "antihyperglycemic" OR "Biguanides"[All Fields] OR "Metformin"[All Fields] OR "Insulin"[All Fields] OR "long acting insulin" OR "intermediate acting insulin" OR "short acting insulin" OR Lente insulin OR Ultralente insulin OR Insulin degludec OR Insulin detemir OR Insulin glargine OR "Insulin zinc suspension" OR Insulin aspart OR Insulin lispro OR Isophane insulin OR "Sulfonylurea"[All Fields] OR Acetohexamide OR Carbutamide OR Chlorpropamide OR Glibenclamide OR Glyburide OR Glibornuride OR Gliclazide OR Glimepiride OR Glipizide OR Gliquidone OR Tolazamide OR Glycopyramide OR "Thiazolidinedione"[All Fields] OR TZD OR Glitazone OR Rivoglitazone OR Pioglitazone OR Rosiglitazone OR "Meglitinides"[All Fields] OR Glinide OR Repaglinide OR Mitiglinide OR "Alpha-glucosidase inhibitors"[All Fields] OR Acarbose OR Miglitol OR Voglibose OR "Dipeptidyl Peptidase 4 Inhibitors"[All Fields] OR DPP-4i OR Alogliptin OR Anagliptin OR Gemigliptin OR Linagliptin OR Sitagliptin OR Saxagliptin OR Teneligliptin OR Vildagliptin OR "GLP-1 Receptor Agonists"[All Fields] OR "GLP-1RA" OR "glucagon-like peptide 1 receptor agonist" OR "glucagon-like peptide 1 agonist" OR Albiglutide OR Dulaglutide OR Exendin 4 OR exenatide OR Liraglutide OR Lixisenatide OR Semaglutide OR Taspoglutide OR "Sodium-Glucose Cotransporter 2 Inhibitors"[All Fields] OR "SGLT-2" OR "SGLT 2" OR "sodium glucose linked cotransporter 2 inhibitor" OR Canagliflozin OR Dapagliflozin OR Empagliflozin OR Ertugliflozin OR Tofogliflozin OR "Pramlintide"	371,533
**#4:** case reports OR meta-analysis OR review	5,450,250
**#5:** Cohort Studies"[Mesh] OR "Randomized Controlled Trial" Filters: humans, excluded preprints	3,213,710
**#6: #1 AND #2 AND #3 NOT #4 AND #5**	49
**EMBASE**	
#1: 'insulin'/exp OR insulin OR 'biguanides'/exp OR biguanidesOR'metformin'/exp OR metformin OR 'sulfonylurea'/exp OR sulfonylurea OR sulfonylure* OR 'acetohexamide'/exp OR acetohexamide OR 'carbutamide'/exp OR carbutamide OR 'chlorpropamide'/exp OR chlorpropamide OR 'glibenclamide'/exp OR glibenclamide OR 'glyburide'/exp OR glyburideOR'glibornuride'/exp OR glibornuride OR 'gliclazide'/exp OR gliclazide OR 'glimepiride'/exp OR glimepiride OR 'glipizide'/exp OR glipizide OR 'gliquidone'/exp OR gliquidone OR 'tolazamide'/exp OR tolazamide OR 'glycopyramide'/exp OR glycopyramide OR 'thiazolidinediones'/exp OR thiazolidinediones OR thiazolidinedione* OR tzd* OR glitazone* OR 'rivoglitazone'/exp OR rivoglitazone OR 'pioglitazone'/exp OR pioglitazone OR 'rosiglitazone'/exp OR rosiglitazone OR meglitinide* OR glinide* OR 'meglitinide'/exp OR meglitinide OR 'repaglinide'/exp OR repaglinide OR netaglinide OR 'mitiglinide'/exp OR mitiglinide OR 'ɑ-glucosidase inhibitors' OR 'alpha glucosidase inhibitor*' OR 'acarbose'/exp OR acarbose OR 'miglitol'/exp OR miglitol OR 'voglibose'/exp OR voglibose OR 'dipeptidyl peptidase 4 inhibitors' OR 'dipeptidyl peptidase iv'/exp OR 'dipeptidyl peptidase iv' OR (dipeptidyl AND ('peptidase'/exp OR peptidase) AND iv AND inhibitor*) OR (('dpp 4i' OR dpp4 OR 'dpp 4'OR (('dpp'/exp ORdpp) AND 4) OR 'dpp iv' OR (('dpp'/exp OR dpp) AND iv)) AND ('inhibitor'/exp OR inhibitor)) OR 'alogliptin'/exp OR alogliptin OR 'anagliptin'/exp OR anagliptin OR' gemigliptin'/exp OR gemigliptin OR 'linagliptin'/exp OR linagliptin OR 'omarigliptin'/exp OR omarigliptin OR 'sitagliptin'/exp OR sitagliptin OR 'saxagliptin'/exp OR saxagliptin OR 'teneligliptin'/exp OR teneligliptin OR 'vildagliptin'/exp OR vildagliptin OR (('glp 1'/exp OR 'glp 1' OR 'glp-1ra' OR 'glucagon-like peptide 1 receptor agonist*' OR 'glucagon-like peptide 1 agonist*') AND ('albiglutide'/exp OR albiglutide)) OR	1,276,516

'dulaglutide'/exp OR dulaglutide OR 'exendin 4'/exp OR 'exendin 4' OR 'exenatide'/exp OR exenatide OR 'liraglutide'/exp OR liraglutide OR 'lixisenatide'/exp OR lixisenatide OR 'semaglutide'/exp OR semaglutide OR 'taspoglutide'/exp OR taspoglutide OR 'sglt 2' OR 'sodium glucose linked cotransporter 2 inhibitor*' OR 'sodium glucose transporter 2 inhibitor*' OR 'sodium glucose cotransporter 2 inhibitor'/exp OR 'sodium glucose cotransporter 2 inhibitor' OR canaglifozin OR 'dapagliflozin'/exp OR dapagliflozin OR 'empagliflozin'/exp OR empagliflozin OR 'ertugliflozin'/exp OR ertugliflozin OR 'tofogliflozin'/exp OR tofogliflozin OR 'pramlintide'/exp OR pramlintide	
**#2:** diabetes OR diabetic* OR 'diabetes mellitus'	1,863,379
**#3:** Parkinson	290,844
**#4:** human	31,024,385
**#5:** review OR 'meta analysis' OR letter	7,947,285
**#6:** [controlled clinical trial]/lim OR [randomized controlled trial]/lim	1,277,462
**#7: #1 AND #2 AND #3 AND #4 NOT #5 AND #6**	**63**
**Cochrane Library**	
**#1:** meglitin(insulin) OR (biguanides OR metformin) OR (sulfonylurea OR sulfonylure* OR acetohexamide OR carbutamide OR chlorpropamide OR glibenclamide or glyburide OR glibornuride OR gliclazide OR glimepiride OR lipizide OR gliquidone OR tolazamide OR glyclopyramide) OR (thiazolidinediones OR thiazolidinedione* or TZD* or glitazone* OR rivoglitazone or pioglitazone OR rosiglitazone) OR (meglitinide* or glinide* OR meglitinide or repaglinide or netaglinide OR mitiglinide) OR (‘α-glucosidase inhibitors’ or ‘alpha glucosidase inhibitor*’ OR acarbose OR miglitol OR voglibose) OR (‘dipeptidyl peptidase 4 inhibitors’ or Dipeptidyl peptidase IV inhibitor* or ((DPP-4i or DPP4 or DPP 4 or DPP IV) and inhibitor) OR Alogliptin OR Anagliptin OR Gemigliptin OR Linagliptin OR Omarigliptin OR Sitagliptin OR Saxagliptin OR Teneligliptin OR Vildagliptin) OR (GLP-1 OR ‘GLP-1RA’ OR ‘glucagon-like peptide 1 receptor agonist*’ OR ‘glucagon-like peptide 1 agonist*’ Albiglutide OR Dulaglutide OR ‘Exendin 4’ or exenatide OR Liraglutide OR Lixisenatide OR Semaglutide OR Taspoglutide) OR (SGLT-2 OR ‘SGLT 2’ OR ‘sodium glucose linked cotransporter 2 inhibitor*’ OR ‘Sodium glucose transporter 2 inhibitor*’ OR ‘Sodium glucose cotransporter 2 inhibitor’ OR Canaglifozin OR Dapagliflozin OR Empagliflozin OR Ertugliflozin OR Tofogliflozin) OR (pramlintide)ide parkinson	
**#2:** diabetes OR ‘diabetes mellitus’ OR diabetic* OR 'diabetes mellitus'	
**#3:** "Parkinson's disease"	
**#4:** human	
**#5:** review AND 'meta analysis' AND letter	
**#6: #1 AND #2 AND #3 AND #4 NOT #5**	**39**

Table S2. Distribution of key effect modifiers across included studies to assess the transitivity assumption

Study	Database	Population	Comparison	Matching	Mean Age (SD/Range)	Sex (% Male)	Cardiovascular Disease (%)	Renal Disease (%)	Follow-up Duration
Brakedal et al. (2017)	Norwegian Prescription Database	Diabetic patients	TZD vs. metformin	PS matching on comorbidities, BMI, drinking status, and time between first DM diagnosis	TZD:62.6(10,7) metformin:64.3(11.6)	TZD: 58.8% Metformin:54.7%	N/A	N/A	3.6 years (IQR 1.7-6.4)
Brauer et al. (2020)	UK THIN database	Diabetic patients (18 years or older)	TZD vs. DPP4i vs. GLP1RA	IPTW matching	TZD:61.9(11.8) DPP4i:63.3(12.5) GLP1RA:56.8(10.7)	TZD:59.6% DPP4i:58.3% GLP1RA:53.9%	**Heart failure**<br>TZD: 2.6% DPP4i: 5.3% GLP1RA: 3.7%<br>**MI**<br>TZD: 5.6% DPP4i: 7.6% GLP1RA: 5.5%<br>**Arrhythmia**<br>TZD: 5.9% DPP4i: 9.3% GLP1RA: 6.3%	TZD:16.7% DPP4i:20.6% GLP1RA:14.6%	6.94 years
Connolly et al. (2015)	Medicare beneficiaries	Older patients (aged ≥65 years) with type 2 diabetes mellitus	TZD vs. sulfonylurea	PS matching on age, sex, race, co-medications, number of hospitalizations, and comorbidities	TZD: 77.63(6.88) sulfonylurea: 77.51(6.89)	TZD: 27.06% Sulfonylurea 27.43%	Atrial fibrillation<br>TZD: 2.87%<br>Sulfonylurea: 3.12%<br>**CHF**<br>TZD: 20.42%<br>Sulfonylurea: 21.01%<br>**CAD**<br>TZD: 35.12%<br>Sulfonylurea: 34.64%	**CKD**<br>TZD: 4.94%<br>Sulfonylurea: 5.30	2.97 years
Guo et al. (2024)	Medicare Part D beneficiaries (15% random sample)	Older patients (aged ≥65 years) with type 2 diabetes mellitus	SGLT2i vs. DPP4i	PS matching on age, number of antidiabetic medications at baseline, comorbidities and year of enrollment	SGLT2i: 73.7 (5.6) DPP4i: 73.7 (5.8)	SGLT2i: 50.13% DPP4i: 49.79%	**Acute MI**<br>SGLT2i 6.6%<br>DPP4i: 6.4%<br>**AFib**<br>SGLT2i:16.5%<br>DPP4i 16.1%<br>**CHF**<br>SGLT2i: 29.4%<br>DPP4i:28.9%	SGLT2i: 66.6% DPP4i: 66.3%	N/A

Huang et al. (2024)	Taiwan's longitudinal health insurance database	Older patients (aged ≥50 years) with type 2 diabetes mellitus diagnosis in	DPP4i vs. sulfonylurea	PS matching on gender, age, income level, and diabetes complications severity index HR adjusted through Bonferroni adjustment test	DPP4i: 61.98 (9.05) Sulfonylurea: 61.94 (9.20)	DPP4i: 54.66% Sulfonylurea: 54.54%	**CAD** DPP4i: 7.55% Sulfonlyurea:8.38% **Arrhythmia** DPP4i: 4.0% Sulfonylurea: 3.83% **HF** DPp4i: 3.08% Sulofnylurea: 2.44%	DPP4i: 1.75 Sulfonylurea: 0.95%	3 years
Mui et al. (2021)	Clinical Data Analysis and Reporting System	T2DM patients with SGLT2i/DPP4i use between January 215 and December 2019	SGLT2i vs. DPP4i	) PS matching on CCI, commodities, non-SGLT2i/DPP4i medications, baseline fasting glucose, and HbA1c	SGLT2i: 61.18 DPP4i: 62.08	SGLT2i: 61.68% DPP4i: 59.19%	**HF** SGLT2i: 1.53% DPP4i: 0.95% **IHD** SGLT2i: 13.45% DPP4i: 8.81% **AFib** SGLT2i: 2.80% DPP4i: 4.98%	SGLT2i: 1.34 DPP4i: 1.68	427 days
Sun et al. (2025)	TriNet X platform (a global federated health research network from 142 healthcare organizations worldwide)	Adults (≥ 18 years) diagnosed with DM and initiated with treatment with either an SGLT2i and metformin without exposure to either drug	Metformin vs. SGLT2i	PS matching on age, sex, ethnicity, comorbidities, and comedications	SGLT2i:66.08 Metformin: 66.24	SGLT2i:57.77% Metformin: 57.82%	**IHD** SGLT2i:41.21% Metformin: 41.31%	N/A	SGLT2i: 987.44±603.2 days Metformin: 944.7±591.5
Tang et al. (2024)	U.S. Medicare administrative data	Older patients (aged ≥66 years) with type 2 diabetes mellitus	GLP1RA vs. DPP4i	stabilized inverse probability of treatment weighting (sIPTW) on age, sex, race, income, comorbidity, and comedication	GLP1RA: 72.7 (5.3) DPP4i: 76.3 (7.2)	GLP1RA:47.3% DPP4i: 44.3%	**AFib** GLP1RA:14.5% DPP4i:19.3% **Acute MI** GLP1RA:5.8% DPP4i:7.3% **CHD** GLP1RA:29.5% DPP4i:36.1% **IHD** GLP1RA:55.8% DPP4i:61.6%	GLP1RA:73.3 DPP4i:71.0%	GLP1RA: 1.54 years (IQR 0.75-2.53) DPP4i: 1.74 (IQR 0.83-2.77)

Zhao et al. (2022)	EMR from Yinzhou Regional Health Care Database (YRHCD),	Adult patients registered in the diabetes registry system and diagnosed with T2DM; or (2) had more than two diagnosis records of T2DM and no records of type 1 diabetes in the electronic medical records. (n=62,400)	AGI vs. TZD	Inverse probability of treatment weighting (IPTW) applied for age, sex, comedications, BMI, cholesterol panel, HbA1C, and medical use	AGI: 60.7 (12.11)<br>TZD: 60.5 (12.05)	AGI: 52.2%<br>TZD: 51.5%	N/A	N/A	2.2 years

Abbreviations: AFib: atrial fibrillation, AGI: alpha-glucosidase inhibitor, CAD: coronary artery disease, CHF: congestive heart failure, CCI: Charlson comorbidity index, CKD: chronic kidney disease, DM: diabetes mellitus, DPP4i: dipeptidyl peptidase-4 inhibitor, GLP1RA: glucagon-like peptide-1 receptor agonist, HF: heart failure, IHD: ischemic heart disease; MI: myocardial infarction, N/A: not applicable, PS: propensity-score, SGLT2i: sodium-glucose co-transporter, T2DM: type 2 diabetes mellitus, TZD: thiazolidinediones,

Table S3. SUCRA table on PD risk across antidiabetics. A) overall analysis and B) sensitivity analysis

A.

Antidiabetic Agents	SUCRA P-value
SGLT2i	0.9333
GLP1RA	0.8445
DPP4i	0.7149
TZD	0.3681
Metformin	0.3597
AGI	0.2112
Sulfonylurea	0.0684

B.

Antidiabetic Agents	SUCRA P-value
GLP1RA	0.9988
DPP4i	0.8043
SGLT2i	0.6969
TZD	0.4990
AGI	0.2619
Metformin	0.2390
Sulfonylurea	0.0001

Table S4. League table of sensitivity analysis

**AGI**	0.26 (0.05–1.39)	0.18 (0.04–1.04)	1.04 (0.19–5.74)	0.33 (0.04–2.53)	0.23 (0.03–1.83)	0.70 (0.21–2.33)
3.90 (0.72–19.69)	**DPP4i**	0.72 (0.31–1.80)	4.08 (0.78–20.85)	1.28 (0.17–10.07)	0.91 (0.27–3.051)	2.74 (0.87–8.58)
5.48 (0.96–26.70)	1.40 (0.56–3.21)	**GLP1RA**	5.70 (0.97–29.06)	1.79 (0.22–13.32)	1.28 (0.28–5.39)	3.84 (1.12–11.68)
0.96 (0.17–5.17)	0.25 (0.05–1.279)	0.18 (0.03–1.027)	**Metformin**	0.32 (0.10–1.037)	0.22 (0.03–1.73)	0.67 (0.20–2.26)
3.06 (0.40–23.28)	0.78 (0.10–5.99)	0.56 (0.08–4.58)	3.18 (0.96–10.33)	**SGLT2i**	0.71 (0.07–7.30)	2.15 (0.40–11.29)
4.28 (0.55–32.99)	1.10 (0.33–3.767)	0.78 (0.19–3.61)	4.49 (0.58–34.88)	1.40 (0.14–15.32)	**Sulfonylurea**	3.00 (0.56–16.37)
1.43 (0.43–4.707)	0.37 (0.12–1.15)	0.26 (0.09–0.90)	1.49 (0.44–5.045)	0.47 (0.09–2.49)	0.33 (0.06–1.80)	**TZD**

Abbreviation: AGI: α-glucosidase inhibitor; DPP4i: dipepidyl-peptidase-4 inhibitor; GLP-1RA: glucagon-like peptide-1 receptor agonist; SGLT2i: sodium-glucose co-transporter-2 inhibitor; TZD: thiazolidinediones
